# Oral Morphine as an Alternative Substitution Treatment for Opioid Use Disorder, a Rare but Non-risk-free Use

**DOI:** 10.3389/fpsyt.2022.893590

**Published:** 2022-06-30

**Authors:** Célian Bertin, Julien Bezin, Chouki Chenaf, Jessica Delorme, Nicolas Kerckhove, Antoine Pariente, Marie Tournier, Nicolas Authier

**Affiliations:** ^1^Université Clermont Auvergne, CHU Clermont-Ferrand, Inserm 1107, Neuro-Dol, Service de Pharmacologie médicale, Centres Addictovigilance et Pharmacovigilance, Centre Evaluation et Traitement de la Douleur, Clermont-Ferrand, France; ^2^Observatoire Français des Médicaments Antalgiques (OFMA)/French Monitoring Center for Analgesic Drugs, Clermont-Ferrand, France; ^3^Institut Analgesia, Faculté de Médecine, Clermont-Ferrand, France; ^4^Bordeaux Population Health Research Center, Team Pharmacoepidemiology, UMR 1219, Inserm, DRUGS-SAFE National Platform of Pharmacoepidemiology, University of Bordeaux, Bordeaux, France; ^5^Service de Pharmacologie médicale, Pôle de Santé Publique, CHU de Bordeaux, Bordeaux, France; ^6^Hospital Charles Perrens, Bordeaux, France

**Keywords:** opioid, morphine, substance use disorder, overdose, opioid maintenance treatment, healthcare database, morphine dependence, prescription medication misuse

## Abstract

**Background:**

National health monitoring agencies have reported the alternative use of morphine sulfate painkiller for maintenance treatment of opioid use disorder (OUD), associated with a potential increase in overdose risk.

**Objectives:**

This study sought to assess the prevalence of regular and occasional legally prescribed morphine use in patients treated for OUD and compare their characteristics to those of patients receiving conventional opioid maintenance treatment (OMT), buprenorphine or methadone. Then, we assessed the factors associated with opioid overdose risk.

**Methods:**

Data were extracted from the French national healthcare system database, covering the entire population in 2015. Diagnosis associated with hospital discharge and long-term disease codes were extracted to select the population and identify outcomes and covariates. OUD non-chronic pain patients were divided into regular (≤35 days between dispensing and ≥3 months of continuous treatment duration) morphine users, and occasional users. Their sociodemographic and health characteristics were compared to OMT controls. A multivariate logistic regression model was performed to determine factors associated with opioid overdose.

**Results:**

In patients treated for OUD, 2,237 (2.2%) morphine users (1,288 regular and 949 occasional), 64,578 (63.7%) buprenorphine and 34,638 (34.1%) methadone controls were included. The prevalence of regular morphine use among patients treated for OUD regularly receiving an opioid was 1.3%. Compared to users who receive morphine regularly, occasional users had an increased risk of overdose [OR = 2.2 (1.5–3.3)], while the risk was reduced in the buprenorphine group [OR = 0.5 (0.4–0.7)] and not significantly different for methadone [OR = 1.0 (0.7–1.4)]. Other overdose risk factors were low-income, comorbidity, i.e., psychiatric conditions, alcohol use disorder or complications related to intravenous drug use, and coprescription with benzodiazepines or pregabalin. These factors were more frequent in morphine groups.

**Conclusions:**

Patients that were prescribed oral morphine represented a small minority of the treated for OUD. The poorer health condition affected by numerous comorbidities and higher risk of opioid overdose in patients treated with oral morphine compared with OMT controls points toward the need to better supervise the practices of these patients, to strengthen multidisciplinary care and risk reduction measures.

## Introduction

Problematic prescription opioid use is a reality for many industrialized countries (1–8) and French national pharmacosurveillance systems have reported the diversion of a specific slow-release pharmaceutical product containing morphine sulfate (MS), named Skenan^®^. This analgesic is available in capsule form, dosed at 10, 30, 60, 100, and 200 mg. It is these lats two highest doses (100 and 200 mg) that are particularly diverted, as confirmed by field studies (9–13). This analgesic is diverted by a minority of patients, sometimes as an occasional illicit drug replacement for heroin, or more regularly in agreement with the prescribing physician, as an alternative opioid maintenance therapy (OMT).

In France, only two medications, buprenorphine and methadone, are approved for the treatment of opioid use disorder (OUD), while MS is only validated as a painkiller. Buprenorphine and morphine can be prescribed by any physician, while methadone can only be prescribed by an addiction specialist. There is currently no real legal framework for the prescription of morphine as an alternative to OMT, which can be prescribed by any physician, regardless of his or her specialty and without restriction regarding the context of care (private practice, primary care, addiction center, or other), nor are there any eligibility criteria well-defined for this treatment. Prescribing MS as an alternative OMT may be justified when the patient reports intolerance or ineffectiveness of conventional OMT (14). This care framework must be identical to that of conventional OMT, with regular medical prescriptions and dispensing in pharmacies. Heroin-alternative MS users report greater availability and quality consistency compared to heroin fluctuations (15). The misused MS then comes either from sporadic medical prescriptions dispensed in pharmacies or from the illicit-market (9–11).

In addition to risks linked to opioid use, notably overdose, there are also those associated with the route of administration. Either as a substitute or alternative to heroin, the MS oral formulation is usually crushed and dissolved to be injected intravenously (10, 11). The alteration of the oral galenic to make it injectable induces risks of thrombosis due to the defective filtration of certain excipients, while the intravenous route presents risks of bacterial and viral complications, both local and systemic (16–22).

A retrospective pharmacoepidemiological study was performed to assess the use of MS prescribed as an alternative OST in patients with OUD and without any chronic pain. The primary study objective was to assess the prevalence of regular and occasional MS use in patients with OUD. The secondary objectives were (i) to compare sociodemographic and health characteristics in patients with OUD treated using MS or conventional OMT, and (ii) to determine the associated factors of opioid overdose.

## Materials and Methods

### Study Design and Data Source

This retrospective descriptive study included patients receiving oral MS, buprenorphine, or methadone in OUD context. It conformed to the RECORD-Pharmacoepidemiological recommendations (23–26).

Data were extracted from the French national healthcare system data (SNDS), often used for public health and pharmacoepidemiological research, between 01/01/2015 and 12/31/2015. SNDS covers 98.8% of the population, comprising exhaustive anonymous individual administrative, medical, and pharmacy data (27, 28). Anonymous identifiers link health reimbursement data, diagnoses codes from hospitalization discharge databases using the 10th revision of the international statistical classification of diseases (ICD-10), and the death registry. Administrative data provides sociodemographic information: year of birth, sex, date of death, free complementary medical cover (CMUc) for low-income status, and any recognized chronic conditions from the list of 30 long-term/major diseases (LTD-30) that are guaranteed full reimbursement for any medical fees. This list is reviewed annually by the government and includes diseases that require particularly costly medical treatment for at least 6 months, like cancer, diabetes, severe heart disorder, chronic psychiatric, neurological or muscular diseases, and chronic lung disease, etc. (29). Pharmacy data comprise anonymous pharmacy identifiers and exhaustive claims for all reimbursed medications dispensed in pharmacies (substances and quantities supplied, dates of prescription, and dispensing) including opioid medications, enabling the daily dosage given to regular users to be estimated. Medical data comprise anonymous doctor identifiers and the specialty of the prescribers.

This study was approved for medical research by the French institute for health data privacy (INDS, no. 176) and the French national data protection commission (CNIL, no. 1946535). French law prohibits the authors from directly sharing the data used for this study, but access can be requested directly from SNDS (website: https://www.health-data-hub.fr).

### Study Population

The criteria used for patient selection were validated by a previous study (30). In accordance with OMT prescription recommendations, we included all men and women aged 15 years and over to whom MS, buprenorphine or methadone was dispensed at least once in 2015. Patients who received regular and concomitant OMT and MS prescriptions were excluded from the analysis due to the inability to link potential complications to either of the two opioids.

OUD patients were identified as:

- having been dispensed buprenorphine or methadone at least once in 2015;
- on the basis of hospital discharge reports or chronic conditions for OUD ICD-10 codes.

All patients diagnosed with cancer or receiving palliative care, as well as patients with chronic pain, were excluded. Patients with chronic pain were identified based on:

- specific ICD-10 codes from hospital discharge reports or LTDs for chronic pain or rheumatic disorders for which MS is recommended in France (31);
- identification of care given in pain clinics;
- identification of continuous analgesic prescription, other than MS, for at least 3 months, considered as the management of chronic pain (32);
- non-affiliation to ‘diagnosis-related groups' who have undergone surgery, to exclude patients who have received MS for post-operative pain.

All ICD-10 codes applied to select the patients are outlined in Supplementary Table 1.

### Medications Exposure

Medications were identified by their Anatomical Therapeutic Chemical (ATC) codes (“N02AA01” and “N02AA51” for morphine alone and in combination, respectively, “N07BC01” for buprenorphine, and “N07BC02” for methadone). Dates of dispensings were used to determine frequency of use.

For MS, buprenorphine and methadone “capsule,” regularity was defined as receiving the medication over at least three consecutive months, during which the treatment was regularly dispensed, i.e., with <35 days between each pharmacy dispensing. This 35-day threshold corresponds to French legal restrictions on opioid medications, which limit their prescription and dispensing to a maximum of 28 days, to which a grace period of 7 days was added in order to avoid overestimating medication discontinuation. Methadone 'syrup' is subject to stricter legislation, with a maximum dispensing period of 14 days, making it necessary to adapt the regularity criterion to 18 days (14 days, plus 4 days of grace period). Patients who fulfilled these criteria were considered regular medication users.

Four groups have been formed. The first comprised all patients with regular MS use in OUD context in 2015. Those who did not fulfill these MS regular-user criteria, but received at least two MS doses in 2015 were included in the second group. The last two groups comprised all OUD control patients receiving regular OMT, separated into buprenorphine on one hand and methadone on the other.

### Study Outcomes and Covariates

All administrative, medical and pharmaceutical data mentioned in “data source” were extracted. All ICD-10 codes applied to identify outcomes and covariates are outlined in Supplementary Table 1.

Diagnosis associated with hospital discharge were extracted to identify unintentional opioid overdoses.

In the same way, diagnosis associated with hospital discharge and LTD codes were extracted to identify the covariates: human immunodeficiency virus (HIV), hepatitis B virus (HBV), hepatitis C virus (HCV), and the main infection complications described as potentially related to intravenous drug injection (16–19). Arterial and venous thrombosis complications (20–22) and various data on comorbidities: severe chronic psychiatric disorders (LTD-23), alcohol use disorders (by ICD-10 code and specific treatments (33), [i.e., disulfiram (“N07BB01”), acamprosate (“N07BB03”), naltrexone (“N07BB04”), and nalmefene (“N07BB05”)], benzodiazepine [anxiolytics (“N05BA”) and hypnotics (“N05CD,” “N05CF”)], and gabapentinoids [pregabalin (“N03AX16”) and gabapentin (“N03AX12”)]) concomitant treatments were also collected. Coprescription was defined as receiving dispensings of the medications involved on exactly the same date, suggesting that the treatments were simultaneously on the same prescription.

Doctor shopping behavior (DSB) was measured in regular morphine, buprenorphine and methadone groups. DSB was defined as a combination of overlapping prescriptions for a specific medication from several different prescribers, dispensed in different pharmacies, to the same patient. This practice enables patients to increase the amount of medications they receive (34, 35) and is typically associated with high levels of misuse and/or diversion (36–40). In this study, the threshold defining a DSB was fixed as:

- at least one day of overlapping prescriptions;
- and at least two different prescribing physicians;
- and at least three different dispensing pharmacies during the study period.

These thresholds correspond to those established by previous studies assessing DSB scores for opioid analgesics, ensuring the comparability of results (41, 42). DSB is measurable only for regular substance use and so is not applicable to the occasional MS group.

The daily opioid dose was calculated for the three regular groups of patients and its oral morphine equivalent was evaluated with a fixed 30:1 ratio for buprenorphine (43) and a validated variable ratio ranging from 4:1 to 12:1 based on the daily dose for methadone (44).

The Charlson Comorbidity Index (CCI), extensively applied in clinical research to account for the confounding influence of comorbidities was calculated (45–47). The CCI assesses the level of comorbidity by considering the level of severity of 19 predefined comorbid disorders, as well as the number of disorders present among them by means of a score (48).

### Statistical Analyses

Categorical variables were expressed as frequencies and associated percentages, and quantitative variables as mean ± standard deviation (SD) or median and interquartile range (IQR), according to their statistical distribution (normality assessed using the Shapiro-Wilk test). The comparison between groups was performed using the chi-squared test for categorical data or Fisher's test where appropriate, with a variance analysis for continuous variables or the Kruskal-Wallis test if normality was rejected.

To determine the influence of various factors associated with overdose in opioid patients, a univariate logistic regression model was performed. The associated *p*-values were computed with their corresponding odds ratios (ORs) and their 95% confidence intervals (95% CI). To study the factors associated with opioid overdose, a multivariate logistic regression analysis was performed. All variables associated with *p* < 0.25 in univariate analysis were included in the model. Age and sex were forced in the model. The corresponding adjusted ORs were calculated with their 95% CIs. All statistical analyses were conducted using SAS-9.4 software (SAS Institute, USA) and STATA-14.2 (StataCorp, USA).

## Results

### Population Description

**Figure 1:**
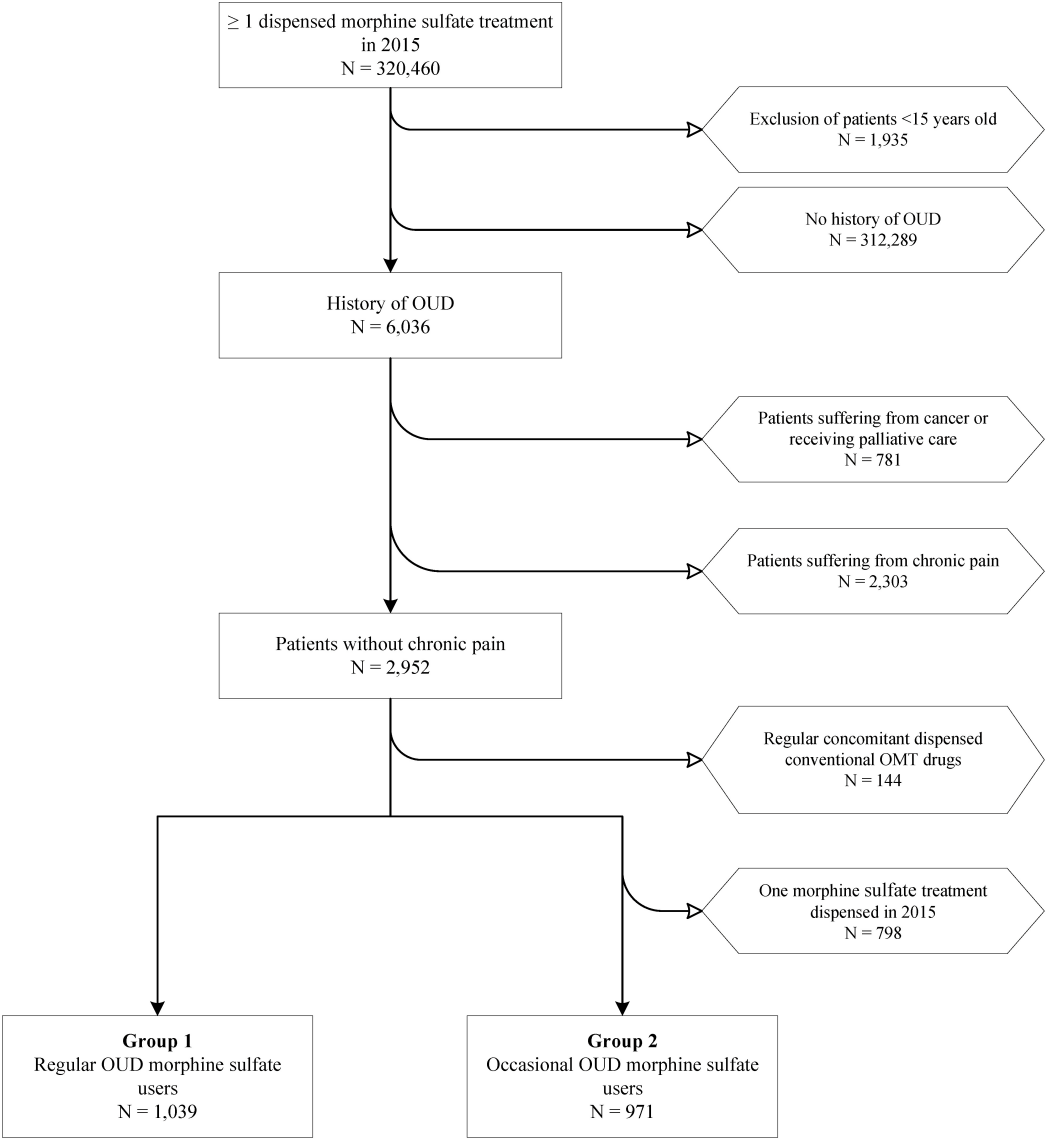
Flow chart of patient selection for interest groups. OUD, opioid use disorder; OMT, opioid maintenance treatment.

From 1 January to 31 December 2015, 101,453 patients with OUD were included, among whom 2,237 patients receiving MS in the context of OUD (2.2%). MS groups were divided between the 1,288 patients who regularly received MS (1.3%), and the 949 who received it occasionally (0.9%) (see flow chart, Figure 1).

A total of 99,216 OMT controls were included in the same period, with 64,578 (63.7%) patients treated with buprenorphine, and 34,638 (34.1%) with methadone (see Supplementary Figure 1). Of the 100,504 OUD patients regularly receiving a regular opioid substitution (MS, buprenorphine or methadone), 1.3% (*n* = 1,288) were regular prescribed MS users.

The study population is described in Table 1. Mean ages were similar in MS and buprenorphine groups; methadone control patients were younger. All groups displayed the same sex ratio, four men to one woman. Over a third of patients receiving MS were considered low-income, based on their CMUc status. The poverty level was higher in regular MS users than occasional ones (*p* < 0.01), and overall, it was higher in MS groups than controls (*p* < 0.01) of which only a quarter benefited from CMUc.

**Table 1:** Characteristics of patients included in the morphine sulfate and control groups.

	**Regular morphine sulfate users with OUD history**	**Occasional morphine sulfate users with OUD history**	**Regular buprenorphine users with OUD history**	**Regular methadone users with OUD history**	***P-*value**
Number	*N* = 1,288	*N* = 949	*N* = 64,578	*N* = 34,638	
Mean age, years ± SD	40.5 ± 10.6	39.8 ± 11.0	39.9 ± 8.7	37.4 ± 8.2	<0.0001
Male sex, % (*N*)	74.8 (963)	78.4 (744)	79.7 (51,480)	76.1 (26,353)	<0.0001
Low-Income status (CMUc), % (*N*)	41.3 (532)	36.0 (342)	27.4 (17,704)	25.5 (8,825)	<0.0001
Overdose, % (*N*)	3.7 (48)	6.9 (65)	1.0 (664)	2.3 (780)	<0.0001
Death, % (*N*)	0.3 (4)	0.7 (7)	0.1 (43)	0.1 (24)	<0.0001
Doctor shopping behavior, % (*N*)	19.9 (256)		3.7 (2,369)	0.8 (280)	
History of HIV, % (*N*)	4.5 (58)	3.8 (36)	1.6 (1045)	1.8 (610)	<0.0001
History of HBV, % (*N*)	1.6 (21)	1.8 (17)	0.6 (396)	0.5 (173)	<0.0001
History of HCV, % (*N*)	27.4 (353)	20.3 (193)	12 (7,716)	12.7 (4,399)	<0.0001
Bacterial infection, % (*N*)	10.5 (135)	10.9 (103)	3.1 (1,995)	2.9 (1,018)	<0.0001
Thrombotic complication, % (*N*)	3.2 (41)	2.7 (26)	0.9 (584)	1.1 (374)	<0.0001
History of psychiatric disorder (LTD-23), % (*N*)	47.9 (617)	44.7 (424)	26.8 (17,301)	34.3 (11,893)	<0.0001
Alcohol use disorder, % (*N*)	25.8 (332)	32.5 (308)	17.8 (11,480)	18.1 (6,285)	<0.0001
Anxiolytic benzodiazepine coprescription, % (*N*)	53.2 (685)	38.9 (369)	37.1 (23,986)	35.5 (12,294)	<0.0001
Hypnotic benzodiazepine coprescription, % (*N*)	32.2 (415)	19.7 (187)	19.7 (12,741)	20.3 (7,013)	<0.0001
Benzodiazepine anxiolytic and hypnotic coprescription, % (*N*)	23.4 (301)	12.9 (122)	12.6 (8,165)	12.8 (4,423)	<0.0001
Pregabalin antiepileptic coprescription, % (*N*)	4.4 (56)	4.1 (39)	0.9 (570)	0.8 (275)	<0.0001
Gabapentin antiepileptic coprescription, % (*N*)	1.1 (14)	0.6 (6)	0.2 (120)	0.2 (58)	<0.0001
Both gabapentinoid coprescription (pregabalin and gabapentin), % (*N*)	0.2 (2)	0 (0)	0 (14)	0 (5)	<0.0001
Opioid daily dose, mg,	443.1 [192.8–758.4]		8.0 [4.1–14.5]	47.7 [28.9–71.8]	
median, [IQR]					
Oral morphine equivalent, mg			240.7 [122.2–435.1]	286.3 [173.5–574.2]	
median, [IQR]					
Coprescription, % (*N*)					
morphine sulfate + OMT ≥ 3 episodes	20.8 (268)	26.2 (249)			

*OUD, opioid use disorder; SD, standard deviation; CMUc, Free complementary medical cover; HIV, Human immunodeficiency virus; HBV, Hepatitis B virus; HCV, Hepatitis C virus; LTD, Long-term/major diseases; IQR, Interquartile range; OMT, Opioid maintenance treatment*.

MS users presented the highest prevalence of psychiatric disorders, with similar rates in both regular and occasional users (*p* = 0.1). Alcohol use disorder was more frequent for occasional than regular MS users (*p* < 0.01) and controls. Control groups were not different (*p* = 0.15). Regular MS users received benzodiazepines coprescriptions more frequently, with rates similar in other groups. Gabapentinoids coprescription rates were higher in MS users than in control groups, which were comparable between them (*p* = 0.5). Regular MS users received gabapentinoids more frequently (pregabalin and/or gabapentin) coprescriptions than occasional ones (*p* = 0.01).

### Outcomes

There were significant differences across all MS patients and OMT control groups (*p* < 0.01) in terms of the prevalence of overdose. Occasional MS patients presented the highest prevalence, followed by regular MS users (*p* < 0.01). The controls were less affected, particularly those taking buprenorphine compared to methadone (*p* < 0.01). Compared to controls (no difference between buprenorphine and methadone, *p* = 0.88), the mortality rate was higher in MS users (*p* < 0.01), and significantly higher for occasional MS users than regular ones (*p* < 0.01).

DSB were significantly higher in regular MS users compared to controls (*p* < 0.01). Buprenorphine controls exhibited significantly higher DSB prevalence than methadone controls (*p* < 0.01).

Compared to OMT controls, between which no difference was found (*p* ≥ 0.1), the prevalences of HIV and HBV infections were significantly higher in MS users, although there was no difference between the regular and occasional groups (*p* ≥ 0.1). It is noteworthy that only the prevalence of HCV infection was different (*p* < 0.01) across all groups, as it was higher among MS (regular > occasional) users compared to controls (methadone > buprenorphine) (*p* < 0.01). The prevalence of bacterial infections was 3.5 times higher in MS groups, *p* < 0.01, with no difference between regular and occasional MS users, *p* = 0.62. The prevalence of thrombotic complications was higher in MS groups than in controls (*p* < 0.01), with comparable prevalence in occasional and regular MS users (*p* = 0.3).

### Characteristics of MS and OMT Prescriptions

The pharmaceutical product Skenan^®^, a sustained-release MS capsule, was ahead of the other prescribed MS forms featuring on 91.3 and 86.9% of prescriptions dispensed to regular and occasional MS users, respectively. The pharmaceutical product Actiskenan^®^, an immediate-release morphine capsule, was the second most frequent MS dispensed to patients with OUD, featuring on 18.8 and 31.5% of prescriptions to regular and occasional MS users, respectively. The pharmaceutical product Moscontin^®^, an extended-release pill, came in third position, featuring in 5.2 and 2.7% of prescriptions dispensed to regular and occasional MS users, respectively (see Supplementary Table 2).

Analysis of Skenan^®^ prescriptions showed a preference for the highest unit doses among regular users, less so among occasional users. For Actiskenan^®^, the dose distribution was more evenly distributed for regular users and low doses were more frequent in the occasional user group (see Supplementary Table 2). Regular MS users presented a median daily dose of 443.1 mg/day [IQR (192.8–758.4)], vs. 8.0 mg/day [IQR (4.1–14.5)] for buprenorphine-treated controls and 47.7 mg/day [IQR (28.9–71.8)] for those on methadone, corresponding to 240.7 mg/day [IQR (122.2–435.1)] and 286.3 mg/day [IQR (173.5–574.2)] equivalent oral morphine, respectively (Table 1).

The prescriptions mainly came from private practice medicine at similar rates among MS patients (regular: 87.4% of prescriptions, occasional: 85.4%) and buprenorphine controls (86.8%), slightly less for methadone controls (67.8%), mainly from general practitioners (GPs) for MS patients (regular: 97.8%, occasional: 97.7%) and similar in control groups (buprenorphine: 98.5%, methadone: 98.1%). Psychiatrists were the second main prescribers, accounting for <1.8% of prescriptions in each group. Occasional MS users more frequently received punctual conventional OMT prescriptions (26.2%) alongside those of MS (≥3 prescriptions/year) than regular MS users (20.8%).

### Factors Associated With Opioid Overdose

In univariate analysis (Supplementary Table 3), general characteristics associated with opioid overdose were young age (*p* = 0.04), low-income (*p* < 0.01), receiving morphine rather than a validated OMT, particularly in the case of occasional MS use (*p* < 0.01), receiving high oral morphine equivalent (*p* < 0.01), and having treatment misuse behaviors according to DSB (*p* < 0.01). Having multiple comorbidities (history of severe chronic psychiatric pathologies, alcohol use disorder and systemic infectious complications (Supplementary Table 1) described as potentially related to intravenous drug injection, arterial, and venous thrombosis complications or according to the CCI score) was significantly associated with opioid overdose risk in univariate analysis. Receiving concomitant benzodiazepines or gabapentinoids and opioid prescriptions was associated with overdose risk in univariate analysis (*p* < 0.01).

Opioid dose in oral morphine equivalent and shopping behavior, reflecting misuse of the medication, were removed from the final logistic regression model because they could not be assessed for occasional MS users.

**Figure 2:**
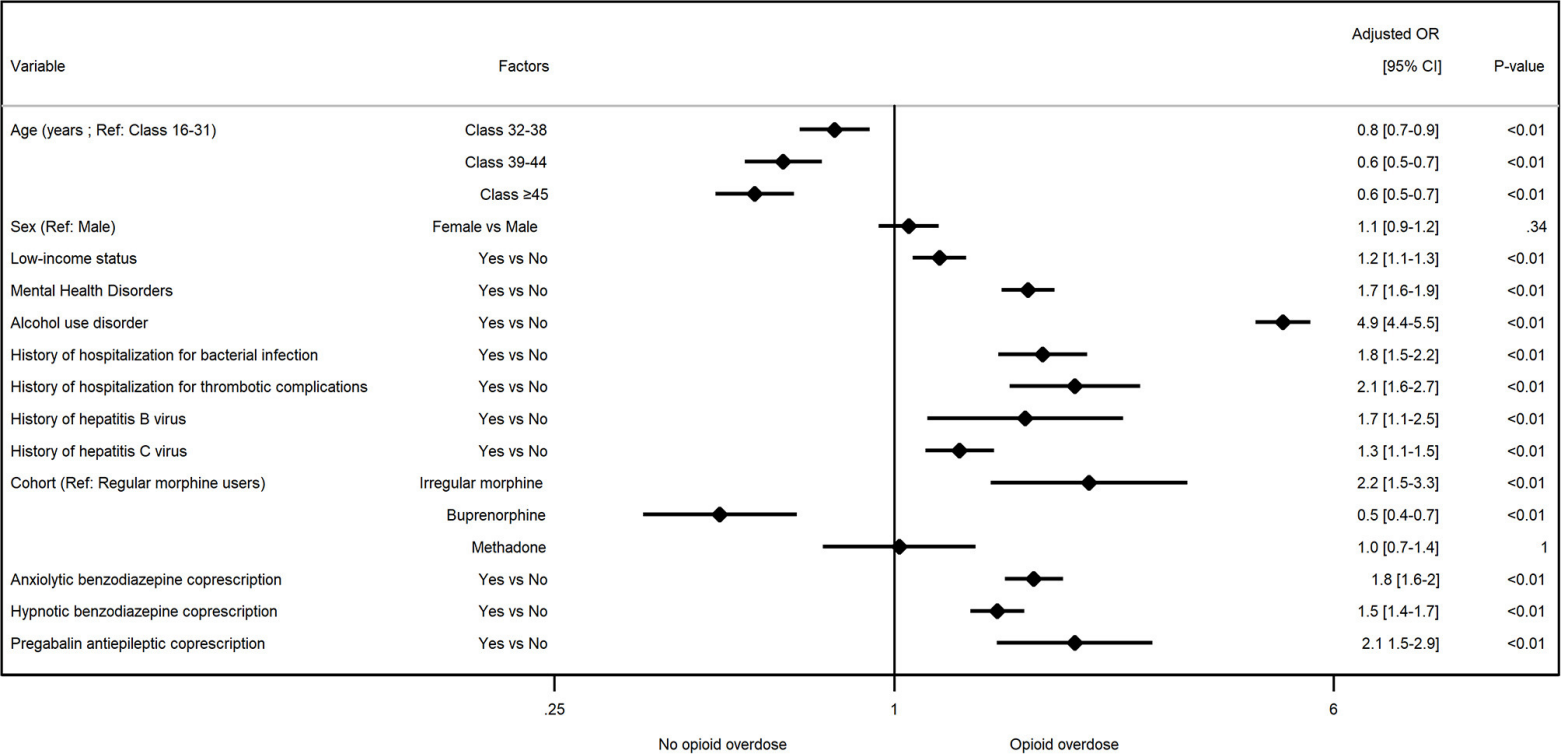
Factors associated with opioid overdose in opioid use disorder patients in multivariate analysis. aOR, adjusted odd ratio.

In the multivariate model, compared to regular MS users, occasional MS users had an increased risk of overdose [aOR = 2.2 (1.5–3.3)], while the risk was reduced in the buprenorphine group [aOR = 0.5 (0.4–0.7)] and not significantly different for methadone [aOR = 1.0 (0.7–1.4)]. When buprenorphine was used as a reference, occasional MS users were at the highest risk of overdose [aOR = 4.5 (3.4–6.0)], followed by methadone controls [aOR = 2.1 (1.9–2.3)], and regular MS users [aOR = 2.0 (1.5–2.8)]. The graphical representation of the resulting multivariate logistic regression model corresponds to the forest plot in Figure 2 (see also Supplementary Table 4). The area under the curve for multivariate model was equal to 0.822 ± 0.18 (Supplementary Figure 2).

Other factors associated with opioid overdose were age, with a risk decreasing over time regardless of the quartile evaluated (*p* < 0.01), being considered low-income [aOR = 1.1 95% CI (1.1–1.3), *p* < 0.01], history of mental health disorders [aOR = 1.7 (1.6–1.9), *p* < 0.01], alcohol use disorder [aOR = 4.9 (4.4–5.5), *p* < 0.01], infection complications described as potentially related to intravenous drug injection [aOR = 1.8 (1.5–2.2), *p* < 0.01], arterial and venous thrombosis complications [aOR = 2.1 (1.6–2.7), *p* < 0.01], HBV [aOR = 1.7 (1.1–2.5), *p* < 0.01], and HCV [aOR = 1.3 (1.1–1.5), *p* < 0.01]. Receiving concomitant benzodiazepines, anxiolytic [aOR = 1.8 (1.6–2.0), *p* < 0.01] or hypnotic [aOR = 1.5 (1.4–1.7), *p* < 0.01], or pregabalin [aOR = 2.1 (1.5–2.9), *p* < 0.01], and opioid prescriptions were associated with an increased overdose risk in multivariate analysis.

## Discussion

In France in 2015, 2,237 patients with OUD were dispensed MS, either regularly or occasionally, i.e., 2.2% of this population. Of the 100,504 patients regularly receiving regular opioid substitution (MS, buprenorphine or methadone) in OUD context, 1.3% (*n* = 1,288) were regular prescribed MS users.

The prevalence of overdoses was the highest in MS users compared to controls. The overdose risk was similar in regular MS users and methadone patients, but higher in these groups than in buprenorphine patients. Compared to buprenorphine controls, occasional MS users had a 4.5 higher risk of overdose, twice that of regular MS or methadone users. This finding seems to indicate that regular MS use, “like a regular conventional OMT,” reduces overdose risk compared to occasional MS use. The known safer pharmacological profile of buprenorphine is also reflected in our results (49, 50).

Several explanations can be given as to why regular opioid use may be more protective against overdose than occasional use. Having regular prescriptions means having regular medical follow-up, allowing better general monitoring of users' health, as well as better management of their comorbidities, which are also overdose associated factors. This regular monitoring also promotes global care, with the adoption of a harm reduction approach associated with treatment.

Finally, having regular prescriptions reduces fluctuations in self-administered doses of opioids (prescribed and possibly illegal). Although they have only irregular dispensing, occasional users still suffer from OUD, implying the onset of a withdrawal syndrome in the absence of regular opioid use. It can therefore be assumed that occasional users continue to use illicit-market opioids in addition to those occasionally prescribed to them, with the variability of self-administered doses that this implies. The stability of self-administered doses may explain the lack of difference in the opioid overdose risk observed between regular MS users and methadone patients.

This risk reduction occurs in patients with regular MS or OMT dispensings despite more frequent gabapentinoid and/or benzodiazepine coprescriptions, possibly for psychiatric comorbidity and/or alcohol use disorder (51–53) which, combined with opioids may increase respiratory depression and overdose risk (53, 54). Although the involvement of benzodiazepines, gabapentinoids, and alcohol in the occurrence of opioid overdose is well-described in the literature (54–58), the highly significant increase in this risk in our multivariate analysis (Figure 2) should attract the attention of practitioners.

All-cause mortality was low in all groups although higher in MS users, probably partially due to their increased overdose rates and the consequences of intravenous injections. In multivariate analysis, the absence of any difference between regular MS and methadone groups in terms of opioid overdose leads to the suspicion that injection behaviors have a significant influence on deaths among MS patients. Therefore, risk reduction measures linked to intravenous injections among these MS users should be reinforced to possibly reduce their risk of death.

Regarding opioid diversion, the regular MS users presented more than a five-fold higher DSB prevalence than controls taking buprenorphine, a substance highly associated with DSB in France (59). The low DSB in methadone controls was consistent with the literature, likely due to the strict monitoring rules imposed on its prescription and dispensing that limit diversion (49, 60). Previous studies have drawn links between DSB, overdose, and death (37, 38, 59, 61), which could partially explain the increased risks in regular MS patients. This high DSB score may show the nomadic nature of certain MS users, but also their difficulty in integrating into our sometimes restrictive care system.

The systemic viral infection rate of MS users (HBV, HCV) and the rate of hospitalization for bacterial infections was, respectively, twice as high and four times greater than those of controls. The prevalence of thrombotic complications in the MS group was also double that of controls, leading us to suspect deficient filtration of excipients when dissolving the oral form for injection (18, 20). These findings are in line with the diversion of oral forms previously described in field studies (9–11) and are linked to opioid overdose in multivariate analysis.

There were more psychiatric comorbidities in MS users, along with higher alcohol use disorder prevalence. The latter was more marked in occasional than in regular MS users. According to previous studies, this may be linked to their increased low-income status (62–67).

Such comorbidities must therefore be systematically investigated and managed in MS users by trained professionals experienced with these dual disorders so common among patients in addiction centers. Moreover, multivariate analysis indicates that these comorbidities, especially alcohol use disorder, are associated with an increase in overdose risk in patients with OUD, in accordance with the literature (68).

These results show that MS prescription for OUD concerns a minority of patients, but suggest that MS exposes them to multiple increased risks compared to conventional OMT. The implementation of a specific care, prescription and dispensing framework would reduce the risk of infection complications, preventing overdose and associated mortality. This care framework should be flexible, so as not to scare off patients with the least stable lifestyles, who are often nomadic, and who change their prescriber depending on their current location. The main objective is to promote a regular “conventional OMT-like” prescription of MS, which appears to involve less risk than occasional use. In the absence of direct access to an addiction center, it might be worthwhile to offer them graduated alternative care. The first level would begin with simply providing risk-reducing tools to involved GPs, or through collaboration with an addiction center. Secondly, MS users could be sent to the GP's addiction center partner to receive multidisciplinary care. Direct or indirect support through the GP for MS prescription by an addictology center would limit the risk of exposing colleagues to the difficulties of caring for these complex patients and would encourage their entry into the multidisciplinary care framework they require. This care setting would be suitable for the eventual provision of an injectable substitution, alone or in addition to a validated OST, whose effectiveness has been scientifically documented (69–71). In various countries (including Switzerland, Netherlands, Germany, Denmark, and Canada), the legal prescription of heroin-assisted treatment under strict supervision has indeed proven to be effective and well-tolerated (69, 72–74).

Results on daily doses (only possible for regular users) confirmed that large MS doses were being taken, in line with already published data (6, 7, 9–11, 75, 76), exceeding those for chronic pain management. This could be a marker of early substance usage disorder (31), which should be a warning sign for prescribers. However, these high dosages are similar to those reported in clinical trials assessing the use of MS as an alternative OMT (77–83). Improving the training of physicians in the identification of OUD and proper prescription rules would promote early detection of these patients and safer use of opioids.

### Strengths and Limitations

These findings should be interpreted by taking into account the strengths and weaknesses inherent in all pharmacoepidemiological studies using healthcare databases. The main risk is that the population included does not resemble that typically encountered in clinical practice. The characteristics of the patients included and controls were similar between groups and consistent with previous field studies (age, sex ratio, low-income status), as were our findings on complications (viral and bacterial infections, DSB) and medication dosages (9–12, 30, 60). This similarity validates our patient selection, despite a selective methodology, and shows the lack of influence of the impossibility to include the 10% of patients receiving their OMT dispensing in an addiction center rather than in pharmacies (9, 10).

Regarding the data source, the SNDS database only provides diagnosis codes attributed on hospital discharge (≥24 h), excluding care provided by emergency services, and we only had access to data on pharmacy dispensing, without details on illicit-market sales. These missing data minimize the size of the groups and the risks to which they are exposed. While our results add to the body of knowledge on opioid overdose and MS use, and are consistent with the results of a previous incidence study on the issue (30), a causal inference cannot be determined directly owing to the cross-sectional design of the study. Further longitudinal studies are needed to explore the temporal relationship between the factors we have identified and opioid overdose. Although oral morphine use disorder seems to be rather specific to France, the main risk factors for opioid overdose do not seem to be specific to this population. The generalization of the study results beyond the French territory must be done with caution and conditioned on future investigations. However, these results may be of interest to countries exposed to the intravenous diversion of oral opioid medications or which wish to offer patients an injectable opioid substitution.

This study also presents significant strengths, notably its ability to recruit a large number of patients for assessing rare issues. This pharmacoepidemiologic approach using a nationwide database is alone in providing sufficient cohort sizes to supply reliable data at the population level, particularly for the patients described here, who are few in number and difficult to follow-up in conventional clinical studies.

## Conclusion

This is the first pharmacoepidemiological study to report the prevalence of 1.3% off-label MS regular users in France among patients regularly receiving OMT for OUD. The high vulnerability and associated comorbidities of these MS users encourage their referral to addiction centers rather than to a GP, so that they can receive multidisciplinary care. Although MS prescription for OUD is considered relevant, it should be accompanied by information on overdose risks and the dispensing of emergency naloxone kits. To reduce the risks associated with the widespread practice of MS intravenous injection, these users should be systematically provided with free sterile injection kits, suitable for this practice, containing large-volume syringes, and special filtering tools (11, 84, 85). The availability of an injectable substitution opioid, self-administered under medical supervision, and ensuring better aseptic conditions, would make it possible to reduce the risks discussed above by promoting access to care for these complex patients.

## Data Availability Statement

The original contributions presented in the study are included in the article/Supplementary Material, further inquiries can be directed to the corresponding author.

## Author Contributions

CB, JD, CC, NA, and JB: conceptualization and methodology. CB and JD: software, data extraction, and formal analysis. CC, JB, and NA: validation and supervision. CB: writing of manuscript. CB, CC, NK, NA, MT, JB, and AP: revision of manuscript. NA: project administration and funding acquisition. All authors contributed to the article and approved the submitted version.

## Funding

This study was part of the MONITOR-OP project (utilisation et sécurité des antalgiques opioïdes en vie réelle), funded by the French National Agency for the Safety of Medicines and Health Products (ANSM) within the framework of the EPI-PHARE scientific interest group—Grant No. 2021S016. This publication represents the views of the authors and does not necessarily represent the opinion of ANSM or EPI-PHARE. The funder had no role in study design, data collection, analysis, interpretation, decision to publish, or preparation of the manuscript.

## Conflict of Interest

The authors declare that the research was conducted in the absence of any commercial or financial relationships that could be construed as a potential conflict of interest.

## Publisher's Note

All claims expressed in this article are solely those of the authors and do not necessarily represent those of their affiliated organizations, or those of the publisher, the editors and the reviewers. Any product that may be evaluated in this article, or claim that may be made by its manufacturer, is not guaranteed or endorsed by the publisher.

## Abbreviations

aOR: Adjusted odd ratio
ATC: Anatomical therapeutic chemical
CCI: Charlson comorbidity index
CI: Confidence interval
CMUc: Free complementary medical cover
CNIL: French national data protection commission
DSB: Doctor shopping behavior
HBV: Hepatitis B virus
HCV: Hepatitis C virus
HIV: Human immunodeficiency virus
ICD-10: International statistical classification of diseases-10th revision
INDS: French institute for health data privacy
IQR: Interquartile range
LTD: Long-term/major diseases
MS: Morphine sulfate
OMT: Opioid maintenance treatment
OR: Odd ratio
OUD: Opioid use disorder
SD: Standard deviation
SNDS: French national system of health data.
