# Supplementary material for: Oral Morphine as an Alternative Substitution Treatment for Opioid Use Disorder, a Rare but Non-risk-free Use
Source: Front Psychiatry. 2022 Jun 30;13:893590. doi: 10.3389/fpsyt.2022.893590 (PMC9282723; doi:10.3389/fpsyt.2022.893590)
Supplement: Supplementary file 1 [file Data_Sheet_1.PDF]

Supplementary Material

Table S1. International classification of diseases (10th revision) selection codes.

	ICD-10 category	ICD-10 codes
Selection of cancer and palliative care	Malignant neoplasms	C
	In situ tumors	D0
	Palliative care	Z515
Selection of chronic pain patients	Chronic pain	R521, R522
	Rheumatologic pain condition	M16, M17, M47, M50, M51, M431, M992, M993, M994, M995
Selection of opioid use disorder patients	Opioid-related disorder	F11
Selection of infectious complications	Human immunodeficiency virus	B20, B21, B22, B23, B24, R75, Z21
	Hepatitis B virus	B16, B180, B181
	Hepatitis C virus	B182, B171
	Cutaneous abscesses	L02, L08, M65
	Phlegmon and cellulitis	L03
	Nasal septum abscess	J340
	Infective endocarditis	I330
	Candida meningitis	B375
	Candida endocarditis	B376
	Candida sepsis	B377
	Other sepsis	A41
	Spondylodiskitis	M463
	Intracranial and intraspinal abscesses	G06
	Endophthalmitis	H440
Selection of thrombotic complications	Venous phlebitis and thrombophlebitis	I80
	Venous post-thrombotic syndrome	I870
	Embolism and venous thrombosis	I828, I829
	Venous disease	I878, I879
	Embolism and arterial thrombosis of the upper limbs	I742
	Embolism and arterial thrombosis of the lower limbs	I743
	Embolism and arterial thrombosis of the distal arteries	I744
	Embolism and arterial thrombosis	I748, I749
Selection of overdose-related hospitalizations	Unintentional opioid intoxication	X42, T400, T401, T402, T403, T404, T406
Selection of alcohol dependence	Alcohol-related disorders	F10

Table S2. Distribution of brand names dispensed, by groups.

	Regular morphine sulphate users with OUD history		Occasional morphine sulphate users with OUD history		Regular buprenorphine users with OUD history		Regular methadone users with OUD history	
Group Number	1 N= 1,288		2 N= 949		3 N= 64,578		4 N= 34,638	
Top Brand Name, % (N)	1- Skenan®	91.3 (1,176)	1- Skenan®	86.9 (825)	1- Subutex®	68.4 (44,146)	1- Syrup	42.4 (14,696)
	200mg	48.6 (572/1,176)	200mg	32.6 (269/825)				
	100mg	60.4 (710)	100mg	41.2 (340)	2- Buprenorphine	42.6 (27,515)	2- Capsule	69.5 (24,062)
	60mg	38.4 (452)	60mg	23.9 (197)				
	30mg	35.5 (417)	30mg	34.5 (285)	3- Suboxone®	6.1 (3,963)		
	10mg	27.9 (328)	10mg	27.8 (229)				
	2- Actiskenan®	18.8 (242)	2- Actiskenan®	31.5 (299)				
	30mg	34.7 (84/242)	30mg	17.1 (51/299)	Distribution		Distribution	
	20mg	36.4 (88)	20mg	23.7 (71)	Subutex® alone	52.7 (34,013)	Capsule alone	57.6 (19,942)
	10mg	48.3 (117)	10mg	60.9 (182)	Buprenorphine alone	27.5 (17,772)	Syrup alone	30.5 (10,576)
	5mg	21.1 (51)	5mg	31.8 (95)	Subutex® + Buprenorphine	13.7 (8,830)	Both	11.9 (4,120)
					Suboxone® alone	3.4 (2,168)		
	3- Moscontin®	5.2 (67)	3- Moscontin®	2.7 (26)	Subutex® + Suboxone®	1.4 (882)		
	4- Sevredol®	0.4 (5)	4- Oramorph®	0.5 (5)	Buprenorphine + Suboxone®	0.8 (492)		
	5- Oramorph®	0.2 (2)	5- Sevredol®	0.4 (4)	Subutex® + Suboxone® + Buprenorphine	0.7 (421)		

OUD: Opioid use disorder.

Table S3. Results of univariate analysis regarding the risk of opioid overdose in opioid use disorder patients.

Associated factors	OR	95%CI	P (Wald)
**Age (quartiles)**			0.04
32-39 *Versus* <32 years	1.0	[0.8-1.1]	
39-45 *Versus* <32 years	0.9	[0.8-1.0]	
≥ 45 *Versus* <32 years	0.8	[0.7-1.0]	
**Sex**			0.13
Femelle *Versus* Male	1.1	[1-1.2]	
**Free complementary medical cover**			<.01
Yes (low-income status) *Versus* No	1.4	[1.3-1.6]	
**Charlson Comorbidity Index**			<.01
Score 1 *Versus* 0	1.7	[1.5-1.9]	
Score 2 *Versus* 0	2.9	[2.1-3.9]	
Score 3 *Versus* 0	4.5	[2.6-7.7]	
Score 4 *Versus* 0	2.8	[1.4-5.8]	
Score 5 *Versus* 0	4.7	[1.5-15.1]	
Score 6 *Versus* 0	1.2	[0.7-2.1]	
Score 7 *Versus* 0	1.5	[1-2.4]	
Score 8 *Versus* 0	2.6	[1.1-6.3]	
Score 9 *Versus* 0	5.7	[1.8-18.4]	
Score 10 *Versus* 0	<0.01	[<0.01->999.9]	
Score 11 *Versus* 0	9.0	[1.1-72.1]	
Score 12 *Versus* 0	<0.01	[<0.01->999.9]	
Score 13 *Versus* 0	<0.01	[<0.01->999.9]	
**Psychiatric disorder**			<.01
Yes *Versus* No	2.9	[2.7-3.3]	
**Alcohol use disorder**			<.01
Yes *Versus* No	7.5	[6.7-8.3]	
**Bacterial infection**			<.01
Yes *Versus* No	3.6	[3-4.2]	
**Anxiolytic benzodiazepine coprescription**			<.01
Yes *Versus* No	3.5	[3.1-3.9]	
**Hypnotic benzodiazepine coprescription**			<.01
Yes *Versus* No	2.7	[2.5-3.0]	
**Benzodiazepine (anxiolytic and hypnotic) coprescription**			<.01
Yes *Versus* No	3.5	[3.1-3.9]	
**Pregabalin antiepileptic coprescription**			<.01
Yes *Versus* No	3.4	[2.5-4.6]	
**Gabapentin antiepileptic coprescription**			<.01
Yes *Versus* No	3.1	[1.6-6.0]	
**Thrombotic complication**			<.01
Yes *Versus* No	4.8	[3.7-6.2]	
**Human immunodeficiency virus**			0.03
Yes *Versus* No	1.4	[1.0-2.0]	
**Hepatitis B virus**			<.01
Yes *Versus* No	3.4	[2.3-4.9]	
**Hepatitis C virus**			<.01
Yes *Versus* No	2.1	[1.9-2.4]	

**Doctor shopping behaviour**			<.01
Yes *Versus* No	2.1	[1.7-2.6]	
**Opioid dose in oral morphine equivalent**			<.01
38.7-252.5 *Versus* 138.7	1.5	[1.3-1.8]	
252.5-480 *Versus* <138.7	1.5	[1.3-1.8]	
≥ 480 *Versus* <138.7	2.6	[2.2-3.0]	
**Group**			<.01
Occasional *Versus* Regular morphine user	1.9	[1.3-2.8]	
Buprenorphine *Versus* Regular morphine user	0.3	[0.2-0.4]	
Methadone *Versus* Regular morphine user	0.6	[0.4-0.8]	

Table S4. Results of multivariate analysis regarding the risk of opioid overdose in opioid use disorder patients.

Associated factors	Ajusted OR	95%CI	P (Wald)
**Age (quartiles)**			<.01
32-39 *Versus* <32 years	0.8	[0.7-0.9]	
39-45 *Versus* <32 years	0.6	[0.5-0.7]	
≥ 45 *Versus* <32 years	0.6	[0.5-0.7]	
**Sex**			0. 35
Femelle *Versus* Male	1.1	[0.9-1.2]	
**Free complementary medical cover**			<.01
Yes (low-income status) *Versus* No	1.2	[1.1-1.3]	
**Psychiatric disorder**			<.01
Yes *Versus* No	1.7	[1.6-1.9]	
**Alcohol use disorder**			<.01
Yes *Versus* No	4.9	[4.4-5.5]	
**Bacterial infection**			<.01
Yes *Versus* No	1.8	[1.5-2.2]	
**Anxiolytic benzodiazepine coprescription**			<.01
Yes *Versus* No	1.8	[1.6-2.0]	
**Hypnotic benzodiazepine coprescription**			<.01
Yes *Versus* No	1.5	[1.4-1.7]	
**Pregabalin antiepileptic coprescription**			<.01
Yes *Versus* No	2.1	[1.5-2.9]	
**Thrombotic complication**			<.01
Yes *Versus* No	2.1	[1.6-2.7]	
**Hepatitis B virus**			<.01
Yes *Versus* No	1.7	[1.1-2.5]	
**Hepatitis C virus**			<.01
Yes *Versus* No	1.3	[1.1-1.5]	
**Group**			<.01
Occasional *Versus* Regular morphine user	2.2	[1.5-3.3]	
Buprenorphine *Versus* Regular morphine user	0.5	[0.4-0.7]	
Methadone *Versus* Regular morphine user	1.0	[0.7-1.4]	

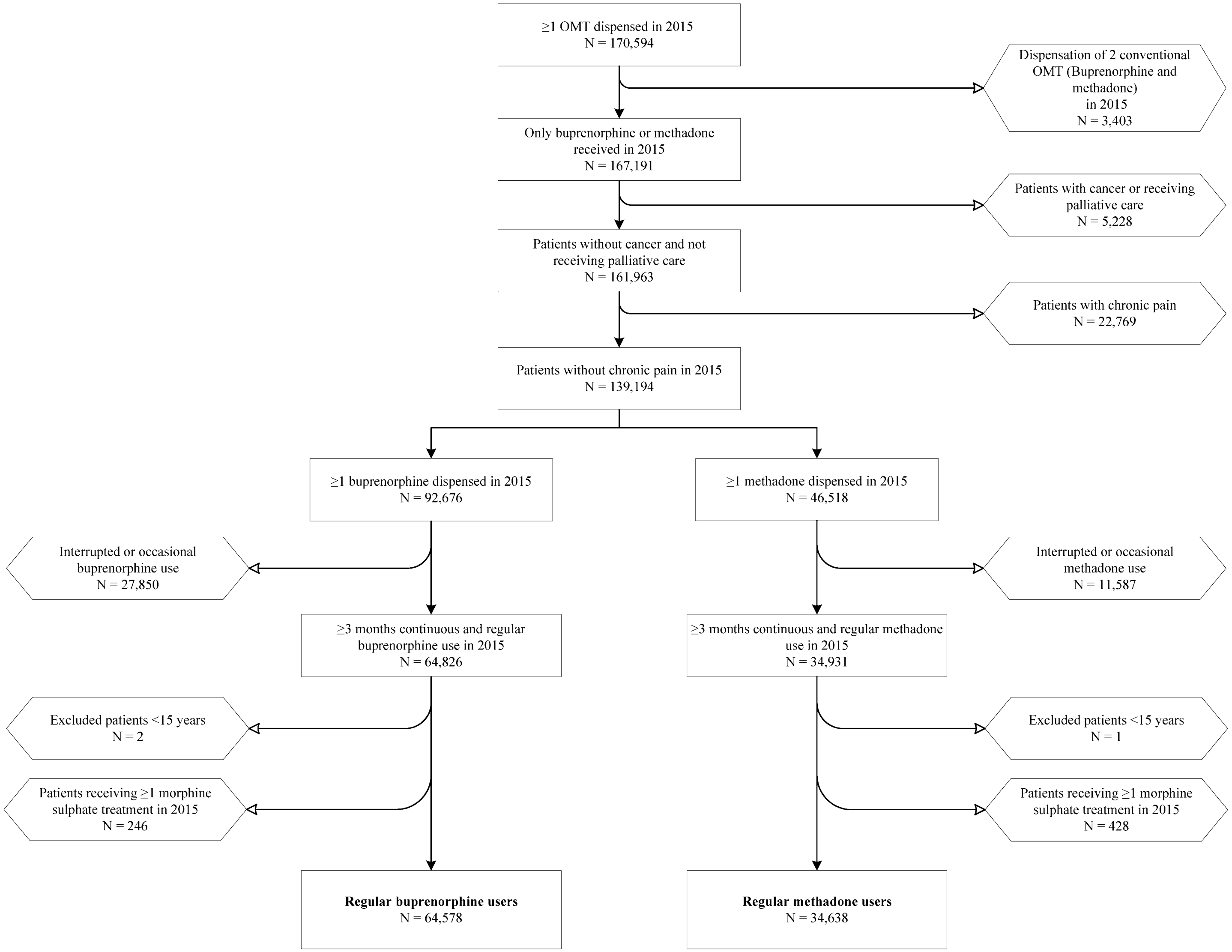

Figure S1. Flow chart of patient selection for control groups.

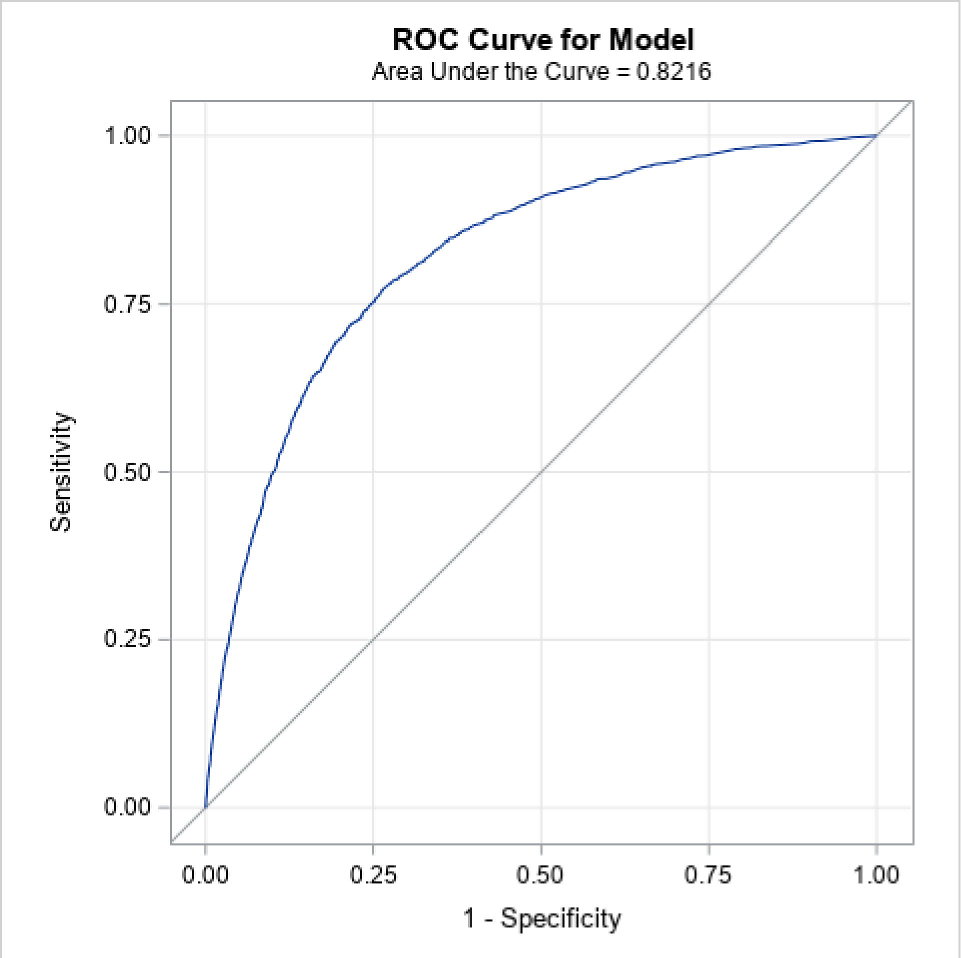

Figure S2. ROC Curve for multivariate analysis regarding the risk of opioid overdose in opioid use disorder patients.
